# Fault-tolerant quantum computing with high-rate symplectic double codes

**DOI:** 10.1126/sciadv.aec4142

**Published:** 2026-11-13

**Authors:** Naoyuki Kanomata, Hayato Goto

**Affiliations:** ^1^ RIKEN Center for Quantum Computing (RQC), Wako, Saitama 351-0198, Japan.; ^2^ Corporate Laboratory, Toshiba Corporation, Kawasaki, Kanagawa 212-8582, Japan.

## Abstract

High-rate and large-distance quantum codes are expected to make fault-tolerant quantum computing more efficient, but most of them lack efficient fault-tolerant encoded-state preparation methods. We propose such fault-tolerant encoders for two symplectic double codes, [[30,6,5]] and [[36,12,5]]. The advantage of these codes is their compactness, namely, their small code block sizes for their high encoding rates and large distance, allowing for early experimental realization of high-rate codes. Detecting crucial errors during encoding with as few auxiliary qubits as possible, our encoders can reduce resource overheads while keeping low logical error rates compared to more naive methods. Numerical simulations with a circuit-level noise model demonstrate the reliability and effectiveness of the proposed methods. We also develop arbitrary-state encoders that enable the injection of arbitrary quantum states into the code spaces. Combined with basic fault-tolerant operations, they support universal quantum computation. We thus demonstrate that efficient and reliable logical state preparation is achievable even for compact and high-rate codes, offering a potential step toward efficient fault-tolerant quantum computing suitable for near-term experiments.

## INTRODUCTION

Quantum error correction (QEC) is a key requirement for realizing large-scale quantum computation. Fault-tolerant quantum computing (FTQC) (*1*–*3*) applies QEC to protect quantum information from noises during computation. So far, the surface code (*4*–*10*) has been the leading candidate for FTQC because of its simple structure and high error threshold. However, one major limitation of the surface code is its low encoding rate, resulting in high resource overheads.

Recently, high–encoding rate quantum codes, such as quantum low-density parity-check (qLDPC) codes (*11*), have drawn growing interest as promising alternatives. These codes may allow us to reduce the overheads associated with FTQC by encoding more logical qubits in a code block. Although these codes are typically more complex than the surface code, the recent progress in experimental systems such as trapped ions (*12*–*15*) and neutral atoms (*16*–*18*) is enhancing the feasibility of their physical implementations.

A major challenge for qLDPC codes is the large block size requirement, which hinders near-term implementations. For example, a typical small-size bivariate bicycle code, which is a leading candidate among qLDPC codes, has parameters [[72,12,6]] (*19*), where [[*n*, *k*, *d*]] means *k* logical qubits encoded into *n* physical qubits and the code distance of *d*. Its modified version called the trivariate (multivariate) bicycle code can shrink the block size to [[30,4,5]] (*20*) while keeping the same error correction ability in terms of code distance. However, its encoding rate becomes lower from 12/72 ≈ 17% to 4/30 ≈ 13%.

In this work, we focus on two Calderbank-Shor-Steane (CSS) codes (*3*, *21*, *22*), [[30,6,5]] and [[36,12,5]], derived from [[15,3,5]] and [[18,6,5]] non-CSS stabilizer codes (*23*), respectively, via so-called symplectic double (*24*). These symplectic double codes have higher encoding rates, 6/30 = 20% and 12/36 ≈ 33%, respectively, than both the above bicycle codes while maintaining error correction ability in terms of code distance. In addition, note that previous studies on FTQC with symplectic double codes have focused on smaller distances up to 3 (*25*).

Toward FTQC using the high-rate symplectic double codes, we propose efficient fault-tolerant zero-state encoders, which are based on the extension of Goto’s minimum-overhead verification method for the Steane seven-qubit code (*26*) to the [[30,6,5]] and [[36,12,5]] codes. We numerically evaluate the performance of the proposed encoders in a circuit-level noise model and show that they can achieve the notable reduction of necessary gates and ancilla qubits while keeping low logical error rates compared to more naive methods.

Alongside designing the zero-state encoders, we also introduce encoders capable of embedding arbitrary quantum states into the code spaces. These arbitrary-state encoders enable universal quantum computation, together with other basic operations through, e.g., so-called magic state distillation (*27*). 

Because of their high encoding rates, small block sizes, and proposed encoders, these symplectic double codes will be useful toward experimental demonstration of low-overhead FTQC in the near future.

## RESULTS

### Definition of the symplectic double codes

In general, symplectic double codes are defined as follows. Consider the stabilizer matrix of a [[*n*, *k*, *d*]] non-CSS code given by

$$H=\left(H_{X}|H_{Z}\right)$$ (1)

where $H_{X}$ and $H_{Z}$ are binary matrices (*3*). Since any two stabilizers must be commutative, the following symplectic orthogonality condition holds:

$$\left(H_{X}\;H_{Z}\right){\left(H_{Z}\;H_{X}\right)}^{T}=H_{X}H_{Z}^{T}+H_{Z}H_{X}^{T}=O$$ (2)

where *O* denotes the zero matrix. In this paper, binary vectors and matrices are defined over GF(2), that is, the addition with them is performed modulo 2. Using this property, we can construct a [[2*n*, 2*k*, *d*]] CSS code by defining the matrices for the *X* and *Z* stabilizers, respectively, as

$$H_{X}^{\prime }=\left(H_{X}\;H_{Z}\right),\;H_{Z}^{\prime }=\left(H_{Z}\;H_{X}\right)$$ (3)

Such a CSS code is referred to as a symplectic double code (*24*).

Hereafter, we focus on the [[30,6,5]] code for simplicity. All the corresponding results for the [[36,12,5]] code are provided in the Supplementary Materials.

For the [[30,6,5]] code, we choose a [[15,3,5]] non-CSS stabilizer code with the following stabilizer matrix (*23*)

$$H=\left(H_{X}|H_{Z}\right)=\left(\begin{array}{ccccccccccccccc} 1 & 0 & 0 & 0 & 0 & 0 & 0 & 0 & 0 & 1 & 0 & 1 & 0 & 1 & 0\\ 0 & 0 & 0 & 0 & 0 & 0 & 0 & 1 & 1 & 1 & 1 & 1 & 0 & 0 & 1\\ 0 & 1 & 0 & 0 & 0 & 0 & 0 & 1 & 1 & 1 & 1 & 0 & 0 & 1 & 0\\ 0 & 0 & 0 & 0 & 0 & 0 & 1 & 1 & 1 & 1 & 1 & 1 & 1 & 0 & 1\\ 0 & 0 & 1 & 0 & 0 & 0 & 0 & 1 & 0 & 0 & 1 & 1 & 1 & 1 & 0\\ 0 & 0 & 0 & 0 & 0 & 0 & 1 & 0 & 1 & 1 & 1 & 1 & 1 & 1 & 1\\ 0 & 0 & 0 & 1 & 0 & 0 & 0 & 1 & 0 & 1 & 0 & 1 & 0 & 0 & 0\\ 0 & 0 & 0 & 0 & 0 & 0 & 1 & 0 & 0 & 1 & 1 & 1 & 1 & 1 & 0\\ 0 & 0 & 0 & 0 & 1 & 0 & 0 & 0 & 1 & 0 & 1 & 0 & 1 & 0 & 0\\ 0 & 0 & 0 & 0 & 0 & 0 & 0 & 1 & 0 & 0 & 1 & 1 & 1 & 1 & 1\\ 0 & 0 & 0 & 0 & 0 & 1 & 0 & 1 & 1 & 0 & 0 & 1 & 1 & 0 & 1\\ 0 & 0 & 0 & 0 & 0 & 0 & 1 & 1 & 1 & 0 & 0 & 1 & 1 & 1 & 0 \end{array}\left|\begin{array}{ccccccccccccccc} 0 & 0 & 0 & 0 & 0 & 0 & 0 & 1 & 1 & 1 & 1 & 1 & 0 & 0 & 1\\ 1 & 0 & 0 & 0 & 0 & 0 & 0 & 1 & 1 & 0 & 1 & 0 & 0 & 1 & 1\\ 0 & 0 & 0 & 0 & 0 & 0 & 1 & 1 & 1 & 1 & 1 & 1 & 1 & 0 & 1\\ 0 & 1 & 0 & 0 & 0 & 0 & 1 & 0 & 0 & 0 & 0 & 1 & 1 & 1 & 1\\ 0 & 0 & 0 & 0 & 0 & 0 & 1 & 0 & 1 & 1 & 1 & 1 & 1 & 1 & 1\\ 0 & 0 & 1 & 0 & 0 & 0 & 1 & 1 & 1 & 1 & 0 & 0 & 0 & 0 & 1\\ 0 & 0 & 0 & 0 & 0 & 0 & 1 & 0 & 0 & 1 & 1 & 1 & 1 & 1 & 0\\ 0 & 0 & 0 & 1 & 0 & 0 & 1 & 1 & 0 & 0 & 1 & 0 & 1 & 1 & 0\\ 0 & 0 & 0 & 0 & 0 & 0 & 0 & 1 & 0 & 0 & 1 & 1 & 1 & 1 & 1\\ 0 & 0 & 0 & 0 & 1 & 0 & 0 & 1 & 1 & 0 & 0 & 1 & 0 & 1 & 1\\ 0 & 0 & 0 & 0 & 0 & 0 & 1 & 1 & 1 & 0 & 0 & 1 & 1 & 1 & 0\\ 0 & 0 & 0 & 0 & 0 & 1 & 1 & 0 & 0 & 0 & 0 & 0 & 0 & 1 & 1 \end{array}\right.\right)$$ (4)

We can verify that the commutativity condition holds

$$H_{X}^{\prime }H_{Z}^{\prime T}=\left(H_{X}\;H_{Z}\right){\left(H_{Z}\;H_{X}\right)}^{T}=O_{12,12}\;$$ (5)

where $O_{i,j}$ denotes the *i* × *j* zero matrix. Thus, we obtain a [[30,6,5]] symplectic double code. One potential drawback of this code is the relatively high weights of its stabilizers, up to 14. This does not fundamentally limit the code’s fault-tolerant properties using Steane- or Knill-style QEC, but it increases the overheads for syndrome extraction, namely, stabilizer measurements. We should take the overheads into account when implementing the present scheme.

The logical Pauli operators for the [[30,6,5]] code are derived as follows. Let $M_{X}$ and $M_{Z}$ denote the binary matrices corresponding to the logical *X* and *Z* operators, respectively. These matrices must satisfy the following commutation and orthogonality conditions:

$$H_{X}^{\prime }M_{Z}^{T}=O_{12,6},\;H_{Z}^{\prime }M_{X}^{T}=O_{12,6},\;M_{X}M_{Z}^{T}=I_{6}$$ (6)

where $I_{i}$ denotes the *i* × *i* identity matrix. The first two conditions ensure that the logical operators commute with all the stabilizers. The final condition ensures the correct commutation and anticommutation relations of the logical *X* and *Z* operators. By finding three vectors orthogonal to and linearly independent of both $H_{X}$ and $H_{Z}$, we obtain the explicit forms of $M_{X}$ and $M_{Z}$ as

$$M_{X}=M_{Z}=\left(\begin{array}{cc} M & O_{3,15}\\ O_{3,15} & M \end{array}\right)$$ (7)

where

$$M=\left(\begin{array}{ccccccccccccccc} 1 & 0 & 0 & 1 & 0 & 1 & 1 & 0 & 0 & 1 & 1 & 0 & 1 & 0 & 0\\ 0 & 1 & 0 & 0 & 0 & 1 & 0 & 1 & 0 & 1 & 0 & 0 & 0 & 1 & 0\\ 0 & 0 & 1 & 0 & 1 & 1 & 0 & 0 & 1 & 1 & 0 & 1 & 0 & 0 & 1 \end{array}\right)$$ (8)

consists of the above three vectors. The logical Pauli operators are expressed more explicitly as follows

$$Z_{L1}=Z_{1}Z_{4}Z_{6}Z_{7}Z_{10}Z_{11}Z_{13}$$ (9)

$$X_{L1}=X_{1}X_{4}X_{6}X_{7}X_{10}X_{11}X_{13}$$ (10)

$$Z_{L2}=Z_{2}Z_{6}Z_{8}Z_{10}Z_{14}$$ (11)

$$X_{L2}=X_{2}X_{6}X_{8}X_{10}X_{14}$$ (12)

$$Z_{L3}=Z_{3}Z_{5}Z_{6}Z_{9}Z_{10}Z_{12}Z_{15}$$ (13)

$$X_{L3}=X_{3}X_{5}X_{6}X_{9}X_{10}X_{12}X_{15}$$ (14)

$$Z_{L4}=Z_{16}Z_{19}Z_{21}Z_{22}Z_{25}Z_{26}Z_{28}$$ (15)

$$X_{L4}=X_{16}X_{19}X_{21}X_{22}X_{25}X_{26}X_{28}$$ (16)

$$Z_{L5}=Z_{17}Z_{21}Z_{23}Z_{25}Z_{29}$$ (17)

$$X_{L5}=X_{17}X_{21}X_{23}X_{25}X_{29}$$ (18)

$$Z_{L6}=Z_{18}Z_{20}Z_{21}Z_{24}Z_{25}Z_{27}Z_{30}$$ (19)

$$X_{L6}=X_{18}X_{20}X_{21}X_{24}X_{25}X_{27}X_{30}$$ (20)

where $X_{Li}$ and $Z_{Li}$ are the Pauli operators for the *i*th logical qubit and $X_{j}$ and $Z_{j}$ are those for the *j*th physical qubit.

For CSS codes, Clifford operations can often be performed fault-tolerantly by so-called transversal gates (*3*). For example, transversal logical CNOT gates can be performed by transversal physical CNOT gates for arbitrary CSS codes.

Next, we consider logical Hadamard gates. By the definitions of $H_{X}^{\prime }$ and $H_{Z}^{\prime }$ in Eq. 3, the transversal physical Hadamard gate followed by the physical SWAP gates between the *q*th and (q+15)th physical qubits (q=1,…,15) exchanges the *X* and *Z* stabilizers, resulting in some logical gates, which are known as Hadamard-type fold-transversal gates based on the *ZX* duality of this code (*25*, *28*). Under the same transformation, the logical *X* and *Z* operators are also exchanged, accompanied by the logical SWAP gates between the *Q*th and (*Q* + 3)th logical qubits (*Q* = 1,2,3), from the definitions of $M_{X}$ and $M_{Z}$ in Eq. 7. In summary, the transversal physical Hadamard gate followed by the physical SWAP gates is equivalent to the transversal logical Hadamard gate, followed by the logical SWAP gates, as shown in Fig. 1.

**Fig. 1.**
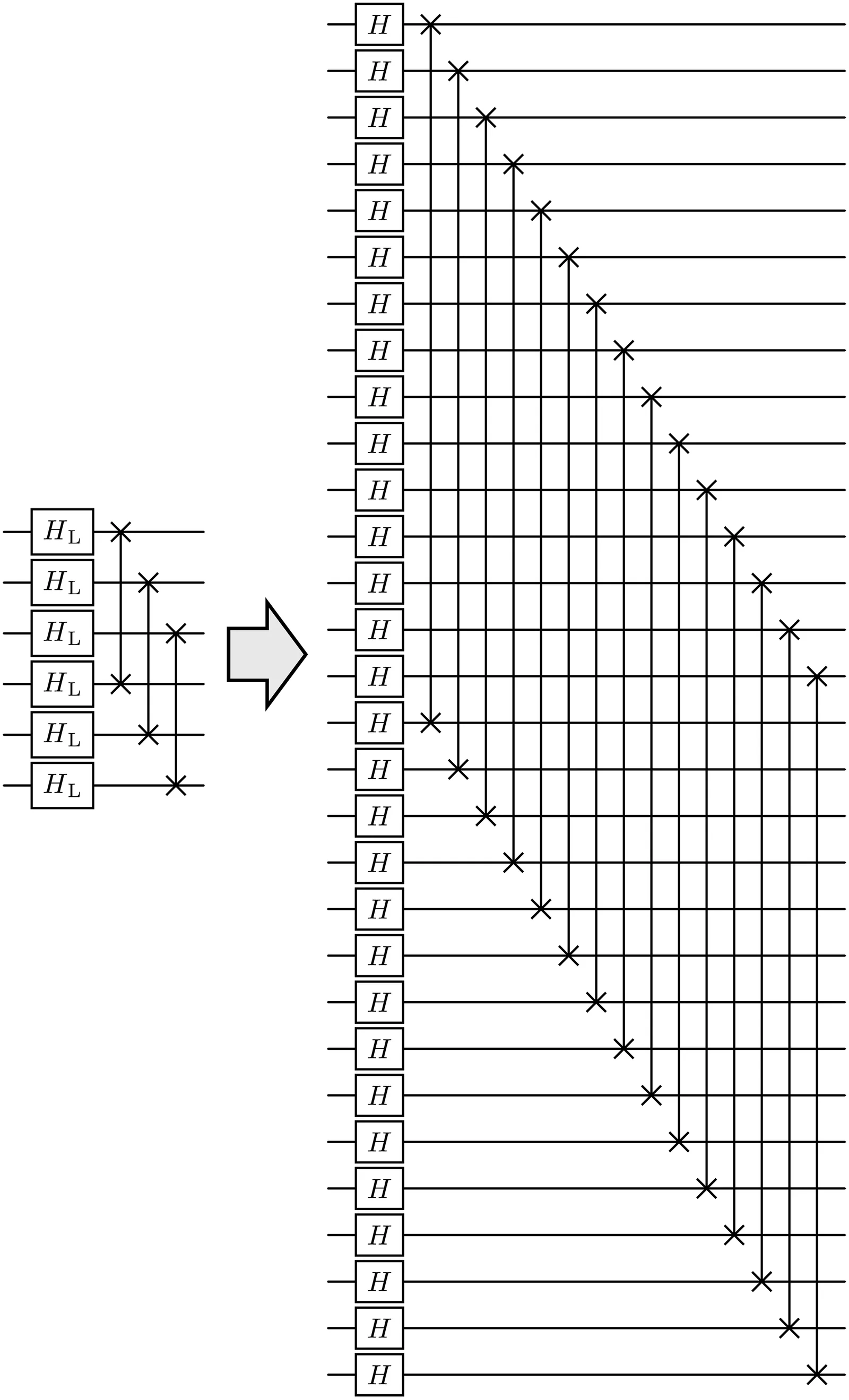
Logical Hadamard gates followed by logical SWAP gates for the [[30,6,5]] symplectic double code.

### Zero-state and plus-state encoders

For the reason mentioned later, here, we first focus on the plus-state encoder of the [[30,6,5]] code, where the plus state is the eigenstate of *X*, namely, $\text{∣+〉=(∣0〉+∣1〉)/}\sqrt{2}$. Using the matrix $H_{Z}^{\prime }$, we can construct the plus-state encoder as follows. By the Gaussian elimination, we first convert $H_{Z}^{\prime }$ to the form such that its left-most part is the identity matrix $I_{12}$. Correspondingly, we prepare the first 12 physical qubits in the eigenstate of *Z*, namely, ∣0〉, and the other 18 qubits in ∣+〉 by Hadamard gates. Then, we perform physical CNOT gates each of which corresponds to a row vector of $H_{Z}^{\prime }$. That is, if the row vector has 1 on the *i*th and *j*th qubits (i≤12 and j>12), then we perform the corresponding CNOT gate whose target and control qubits are the *i*th and *j*th qubits, respectively. Thus, the 30-qubit state lastly becomes the stabilizer state, namely, the simultaneous eigenstate with eigenvalues of +1, with respect to not only all the *Z* stabilizers but also all the *X* stabilizers and all the logical *X* operators, which is the logical all-plus state of the [[30,6,5]] code. The resultant plus-state encoder is shown in Fig. 2.

**Fig. 2.**
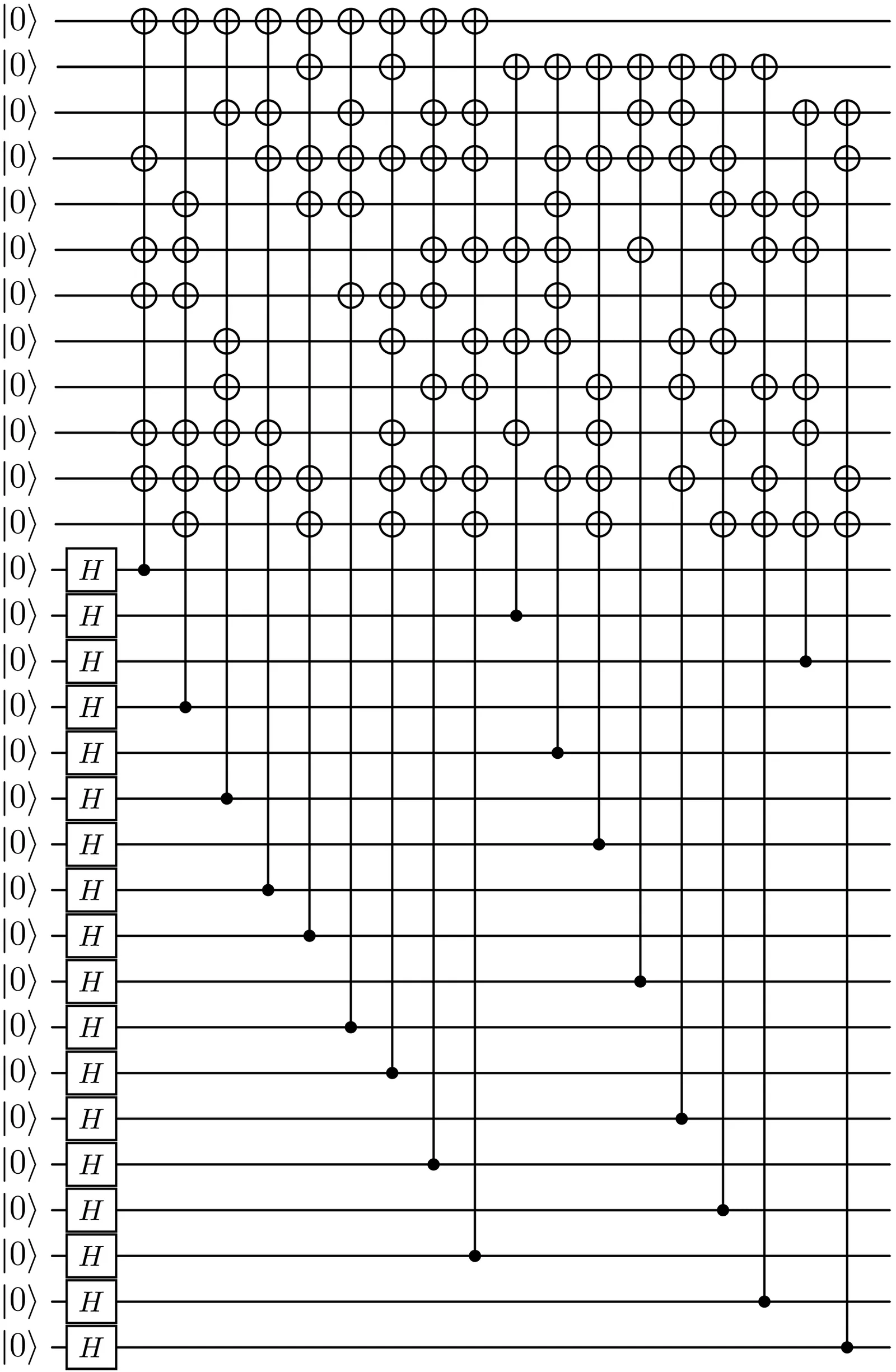
Plus-state encoder for the [[30,6,5]] symplectic double code with 108 CNOT gates.

The above encoder needs 108 CNOT gates. To reduce them, we next apply the overlap method proposed in (*29*) to the encoder. The point of the overlap method is as follows. If two column vectors, e.g., the *j*th and *l*th column vectors of $H_{Z}^{\prime }$ (j,l>12) have two or more overlaps in terms of 1, then there is a possibility for reducing the number of CNOT gates by performing a CNOT gate on the *j*th and *l*th qubits, instead of the above CNOT gates on them. Optimizing these CNOT gates, we succeeded in reducing the number of CNOT gates from 108 to 67, as shown in Fig. 3.

**Fig. 3.**
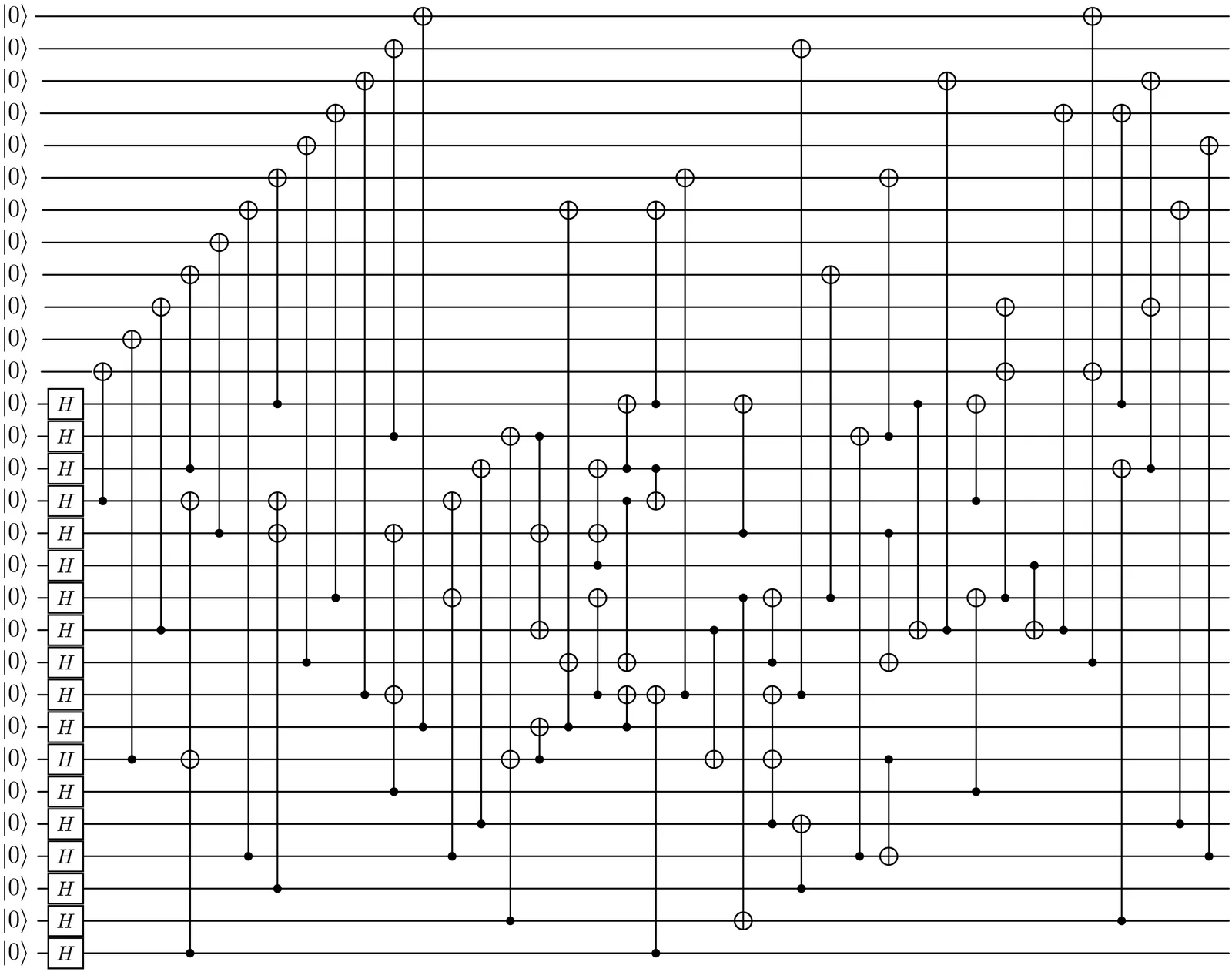
Plus-state encoder for the [[30,6,5]] symplectic double code with 67 CNOT gates.

In a similar manner to the plus-state encoder in Fig. 3, we can construct the zero-state encoder using $H_{X}^{\prime }$ and the overlap method. However, the logical all-zero state obtained by such an encoder exhibited lower performance than the logical all-plus state obtained by the encoder in Fig. 3 under evaluation with a circuit-level noise model used in the “Simulation of logical CNOT gates” section. (The performance difference may come from our specific choice of the qubits for the Gaussian elimination, such that the left-most part of a check matrix is converted into the identity matrix.) This is the reason why we started with the plus-state encoder. Instead of using $H_{X}^{\prime }$, we can prepare the all-zero state using the plus-state encoder, followed by the logical Hadamard and SWAP gates shown in Fig. 1, resulting in another zero-state encoder. We use it as a zero-state encoder throughout this work.

### Decoder

In this work, we developed a decoder based on a lookup table specifically designed for the [[30,6,5]] symplectic double code. This is achievable because of its compact structure, where the “compactness” means that the number of syndromes used for decoding is only 12, resulting in only 2^12^ rows of the lookup table. (It has turnded out that we must consider error patterns up to weight 4 to complete the 2^12^ rows.) This decoder is optimal in the sense that the syndrome information is fully used.

The lookup table for the logical *X* measurement is built as follows. First, we generate all possible phase-flip error patterns up to weight 4, and compute the corresponding syndromes as follows

$$s_{j}=H_{X}^{\prime }e_{j}$$ (21)

where $e_{j}$ and $s_{j}$ are the binary vector and syndrome values, respectively, corresponding to the *j*th phase-flip error pattern. If different error patterns give the same syndrome value, then we choose the lowest-weight one. Thus, every syndrome value and its associated error pattern are cataloged, resulting in the lookup table for the logical *X* measurement. The lookup table for the logical *Z* measurement can also be constructed with $H_{Z}^{\prime }$, instead of $H_{X}^{\prime }$, in a similar manner.

The table provides the most likely error pattern for a syndrome result. The decoder uses the table to find the most likely error pattern, where the syndrome result is used as the index of the error pattern.

We evaluate the performance of our decoder by numerical Monte-Carlo simulations, where we assume the code capacity noise model, that is, there are only physical-qubit memory errors and no other kinds of errors. [Such code capacity experiments can be done by generating an error vector and decoding it with the code’s parity-check matrix. However, we use stabilizer simulations to verify our proposed encoding circuit. In this work, we used Stim (*30*) for stabilizer simulations]. We first create an error-free logical all- plus state of the symplectic double code, utilizing the encoder in Fig. 3. Then, independent phase-flip errors are applied to physical qubits, with probability of $p_{\text{flip}}$. Following that, we carry out ideal measurements on all the physical qubits in the *X* basis. We decode these outcomes using the lookup table explained above. We consider that the decoding has succeeded if all the decoding outcomes for the six logical qubits are ∣+〉 and, otherwise, it has failed. The decoding error probability was estimated through 112,000,000 trials for $p_{\text{flip}}=0.001$ and 11,200,000 trials otherwise.

Figure 4 shows the results of the decoding error probability as a function of $p_{\text{flip}}$. The decoding error probability follows a power law proportional to $p_{\text{flip}}^{2.91}$. The exponent 2.91 is in reasonable agreement with the theoretically expected value based on the code distance 5, namely, (5 + 1)/2 = 3, indicating that the decoder is optimal, as expected. [A distance 5 code can correct all weight 2 errors and cannot correct some weight 3 errors, resulting in $\Theta \left(p_{\text{flip}}^{3}\right)$ logical error probability].

**Fig. 4.**
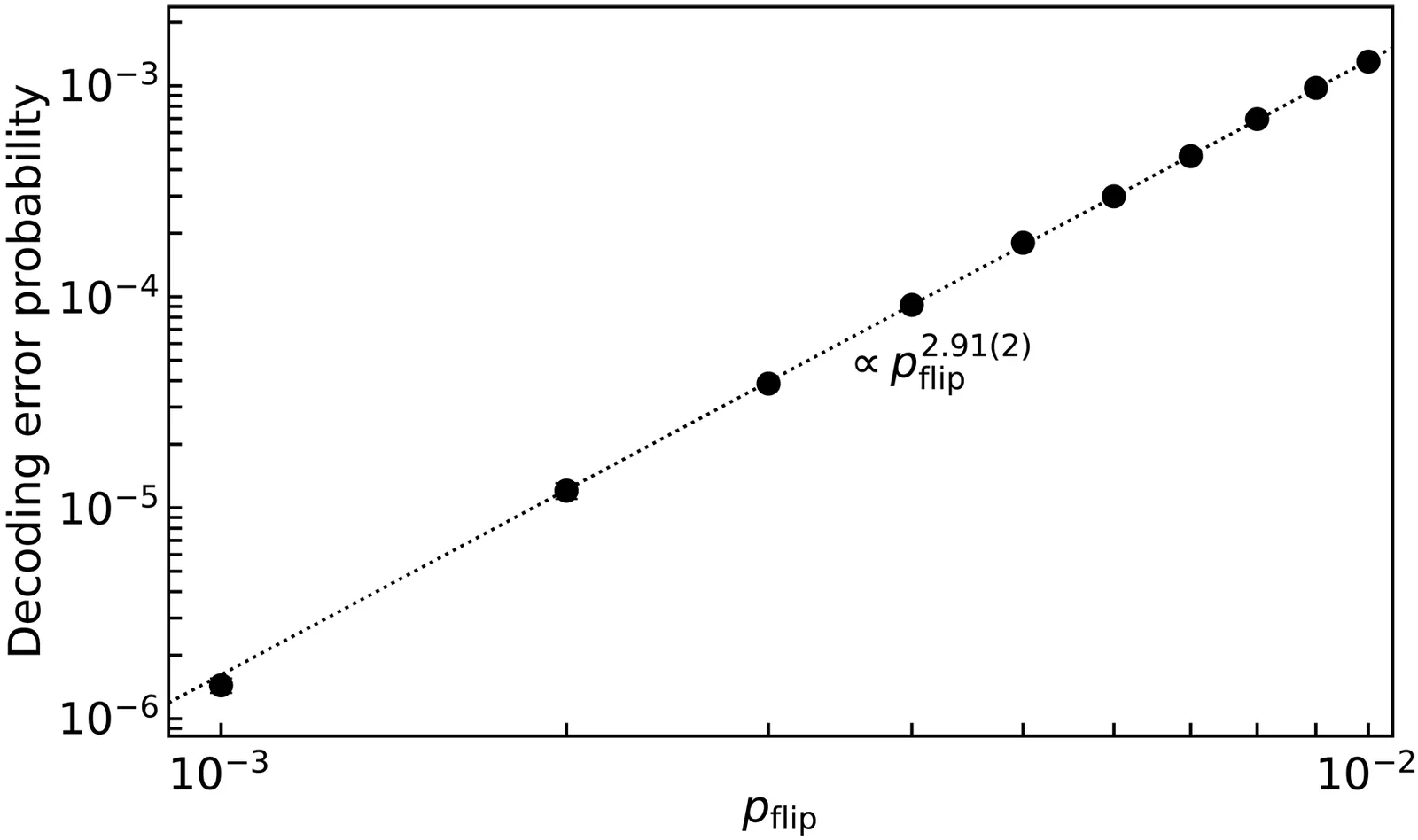
Simulation results of the decoding error probability for the [[30,6,5]] symplectic double code under phase-flip (*Z*) errors with probability of *p*_flip_.

### Fault-tolerant zero-state and plus-state encoders

Note that the encoders presented in the “Decoder” section are not fault tolerant because of correlated multiqubit errors due to CNOT gates. Here, we make the encoder in Fig. 3 fault-tolerant by generalizing Goto’s minimum-overhead verification method for the Steane code (*26*). This method achieves fault tolerance by first preparing a logical state using a non–fault-tolerant encoder and then eliminating potentially harmful errors through appropriate logical Pauli measurements. Here we focus on a fault-tolerant encoder for the all-plus state. As explained in the “Decoder” section, the logical all-zero state can be prepared by transversal Hadamard gates on the all-plus state, which is fault-tolerant.

We first rearrange the physical CNOT gates in Fig. 3 so that the instantaneous state at each step is a stabilizer state with as low stabilizer weights as possible. Next, we find a special step at which all the stabilizer weights are less than or equal to 3, but some of them exceed 3 at the next step. Such low-weight stabilizers allow us to regard the correlated errors as single-qubit errors (*26*, *31*), and, therefore, we care about only the correlated errors after the special step, similarly to “hook” errors in the surface-code syndrome measurements, where only correlated errors due to the third and fourth CNOT gates are harmful (*5*).

Next, we search for logical *X* measurements that can catch all the correlated errors caused by CNOT gates after the above special step, where we minimize the number of the logical *X* measurements and the weights of the logical *X* operators. This search results in the verification circuit in Fig. 5A. We accept the output state if all the measurement outcomes are zero.

**Fig. 5.**
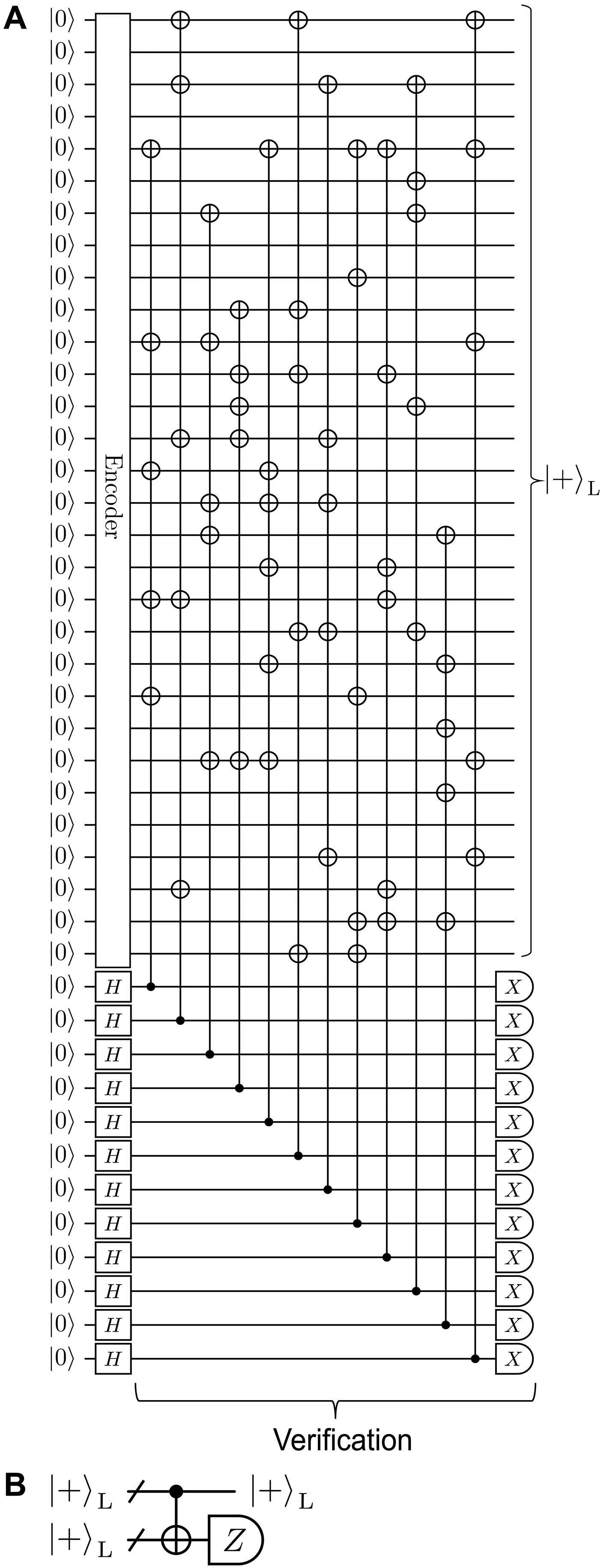
Fault-tolerant plus-state encoder for the [[30,6,5]] symplectic double code. (**A**) Plus-state encoder using Goto’s verification method (*26*) to eliminate harmful *Z* errors. The “Encoder” box is the quantum circuit shown in Fig. 3. (**B**) Verification procedure to eliminate harmful *X* errors. The two input states are prepared by the encoder in (A).

While the above verification can eliminate harmful *Z* errors, we also have to eliminate harmful *X* errors. To achieve that, we use the circuit in Fig. 5B, where we first prepare two logical all-plus states using the circuit in Fig. 5A, then perform transversal logical CNOT gates, and measure the second logical block in the *Z* basis. We accept the logical state in the first block if no errors are detected, that is, if all the stabilizer and logical values of the measurement outcomes are zero. Our proposed fault-tolerant plus-state encoder thus consists of the circuits in Fig. 5, A and B. For comparison, we also evaluate more naive fault-tolerant encoders shown in Fig. 6 (A to C), where many copies of all-plus states are prepared with the encoder in Fig. 3 and harmful *Z* and *X* errors are eliminated by verification with the copies (*32*).

**Fig. 6.**
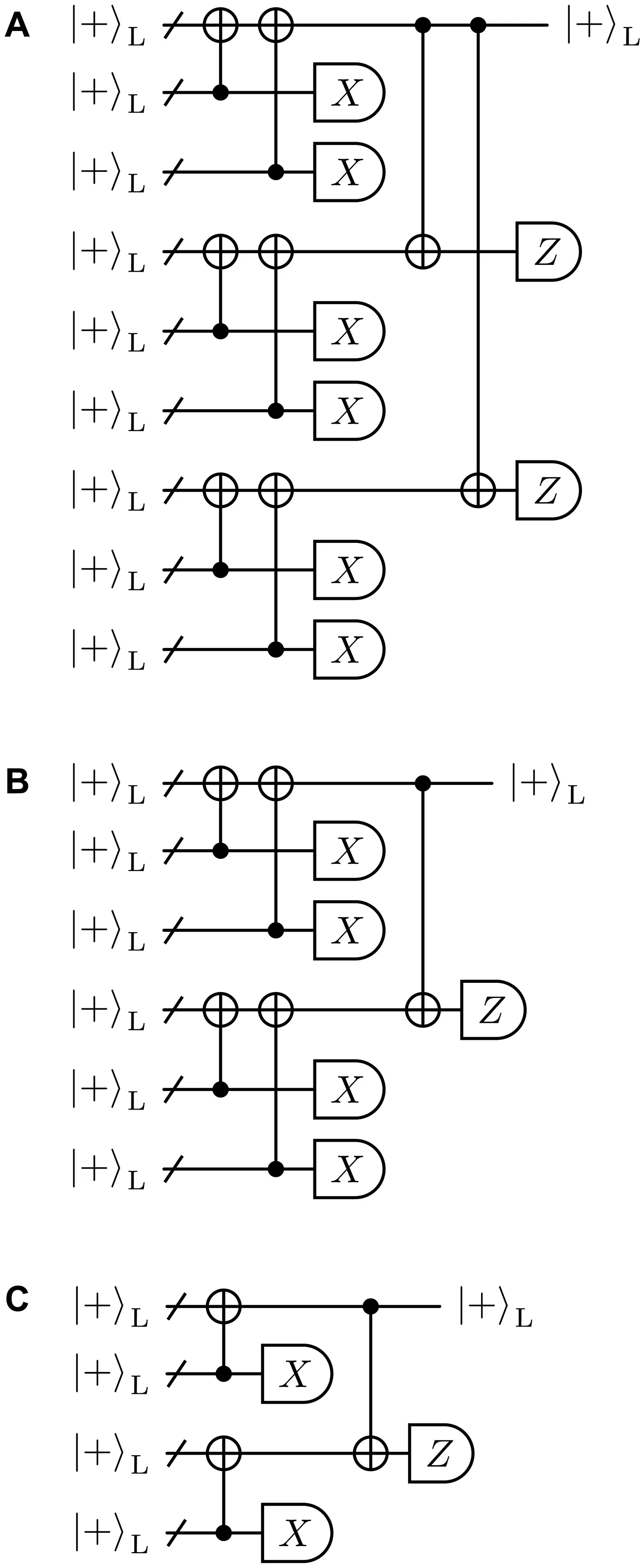
Naive fault-tolerant plus-state encoders. (**A**) Nine-copy encoder. We use three copies for the verification to eliminate both *Z* and *X* errors. The input copies are prepared by the encoder in Fig. 3. We accept the output state if no error is detected, otherwise restart the encoding. (**B**) Six-copy encoder. We use three copies for the verification to eliminate *Z* errors and two copies for *X* errors. (**C**) Four-copy encoder. We use two copies for the verification to eliminate both *Z* and *X* errors.

### Simulation of logical CNOT gates

Here we evaluate the performance of the encoders presented in the “Fault-tolerant zero-state and plus-state encoders” section through numerical simulations of logical CNOT gates. The simulation method is depicted in Fig. 7A (*33*), where faulty transversal logical CNOT gates followed by QEC gadgets (*34*–*36*) are performed on two ideal logical Bell states. In this work, we perform QEC by error-correcting teleportation depicted in Fig. 7B. After disentangling the Bell states and measuring all the qubits, the outcomes are decoded with the lookup tables.

**Fig. 7.**
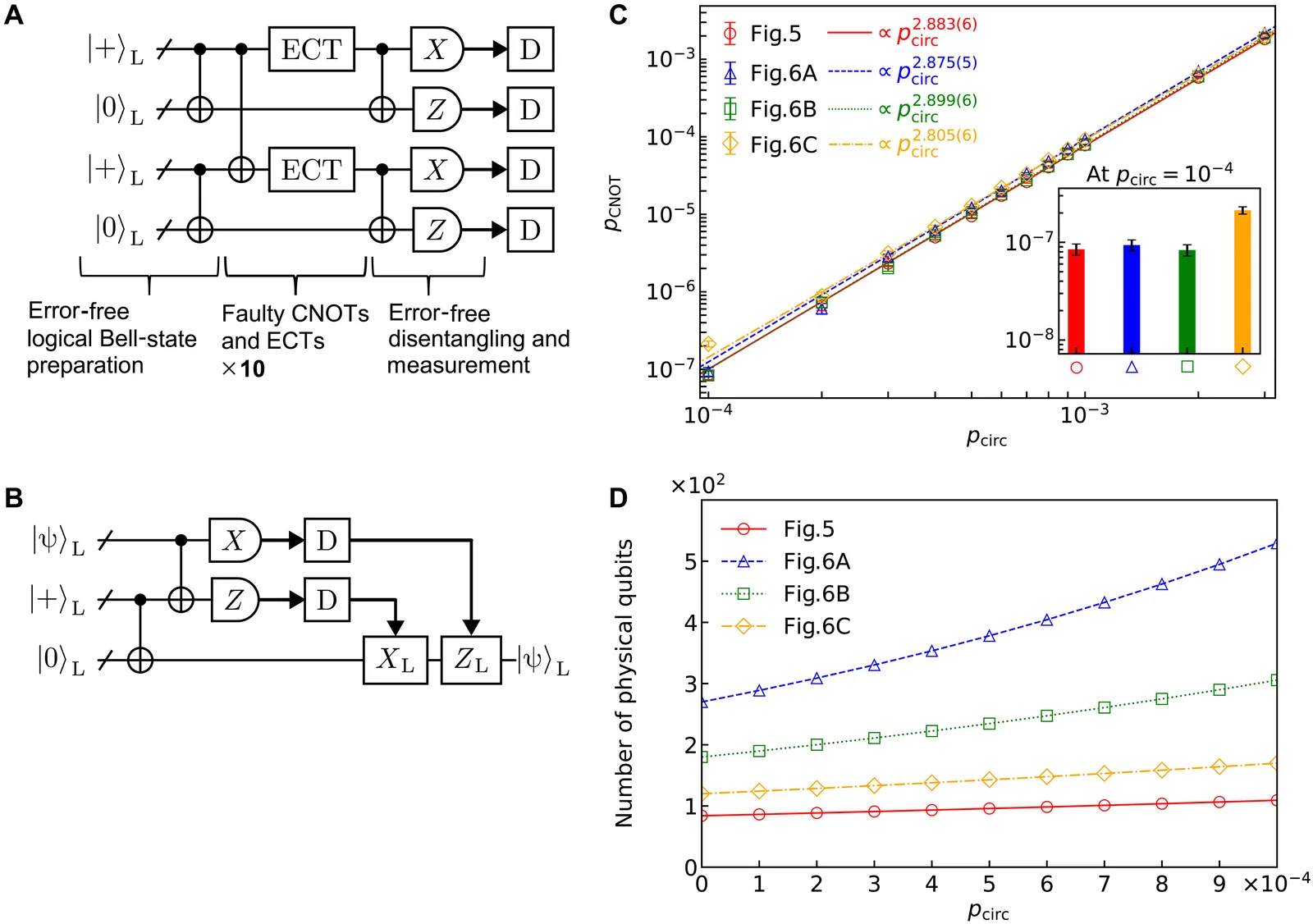
Evaluation of the encoders and logical CNOT gates for the [[30,6,5]] symplectic double code. (**A**) Simulation method for estimating logical-CNOT error probability $p_{\text{CNOT}}$ (*33*). We first create two error-free logical Bell states encoded with the [[30,6,5]] symplectic double code. On the Bell states, we perform 10 sets of transversal logical CNOT gates, followed by QEC gadgets, which are implemented by faulty transversal physical CNOT gates and error-correcting teleportation (ECT) (*34*–*36*), respectively. Following this, the logical states are disentangled with error-free CNOT gates. Finally, all physical qubits are ideally measured in the *Z* and *X* bases. Measurement outcomes are decoded with the lookup tables. If all decoded logical outputs are consistent with the input states, then we consider that the ten transversal logical CNOT gates have succeeded and, otherwise, they have failed, the failure probability of which is denoted by $p_{10}$. The logical CNOT error probability is evaluated as $p_{\text{CNOT}}=1-{\left(1-p_{1}\right)}^{1/6}$ with $p_{1}=1-{\left(1-p_{10}\right)}^{1/10}$. (**B**) Error-correcting teleportation (*34*–*36*) used in (A). The logical Bell states for error-correcting teleportation are prepared with one of the encoders in Figs. 5 and 6 in the circuit-level noise model described in the main text, where the preparation of two logical all-plus states used for the logical Bells state is repeated until success, namely, passing the verification stage with no detected errors. (Memory errors during waiting time until success are not taken into account in this work.) The CNOT gates and measurements for the logical Bell measurement are also faulty. (**C**) Numerical results of the logical-CNOT error probability as a function of the error rate, $p_{\text{circ}}$, of the noise model. (**D**) Average total number of physical qubits required for preparing a logical all-plus state passing the verification stage with no detected errors.

In this work, we apply the following circuit-level noise model (*33*) to the faulty logical CNOT gates and QEC gadgets. The error rate is denoted by $p_{\text{circ}}$. Each physical CNOT gate is implemented by an ideal CNOT gate, followed by one of 15 nonidentity two-qubit Pauli errors with probability of $p_{\text{circ}}/15$. Each physical-qubit preparation in ∣0〉 is followed by a bit-flip error with probability of $p_{\text{circ}}$. Before each physical-qubit measurement in the *Z* basis, a bit-flip error occurs with probability of $p_{\text{circ}}$. We assume no memory and single-qubit gate errors. This error model may be relevant for some physical systems such as trapped ions (*12*–*15*) and neutral atoms (*16*–*18*).

Figure 7C shows that the logical-CNOT error probability $p_{\text{CNOT}}$, which was estimated through 11,200,000 trials for $p_{\text{circ}}=0.0001$ and 1,120,000 trials otherwise, follows a power law as $p_{\text{CNOT}}\propto p_{\text{circ}}^{a}$. For all the encoders, the estimated values of the exponent *a* are close to 3, which is consistent with the code distance of 5. This demonstrates the fault tolerance of the encoders. Moreover, the proposed encoder achieved the lowest $p_{\text{CNOT}}$ at $p_{\text{circ}}=10^{-4}$, as shown in the inset in Fig. 7C. The logical-CNOT error probability of 10^−7^ at $p_{\text{circ}}=10^{-4}$ is much lower than that for the distance 3 Steane code, which is estimated at 10^−5^ (*26*). This shows the advantage of the large-distance symplectic double codes. (The logical-CNOT error probabilities for the [[36,12,5]] code are comparable to those for the [[30,6,5]] code; see the Supplementary Materials for details.)

Additionally, we numerically estimated the average total number of physical qubits required for the preparation of a logical all-plus state passing the verification stage with no detected errors, where the repetition due to postselection is taken into account. The results are shown in Fig. 7D, which shows that the proposed encoder in Fig. 5 can achieve not only remarkably fewer physical qubits but also a smaller slope, namely, higher acceptance probability than the naive encoders in Fig. 6 (A to C), as expected. The small slope indicates the small impact of the postselection in the proposed encoder.

### Arbitrary-state encoder

To achieve universal fault-tolerant quantum computation, e.g., via magic-state distillation (*27*), we need an arbitrary-state encoder to inject a magic state into the code space. For this purpose, here, we propose an arbitrary-state encoder for the [[30,6,5]] symplectic double code. (Note that this encoder is not fault-tolerant. Therefore, to achieve fault tolerance, we need appropriate verification like magic state distillation after logical-state preparation using this encoder.)

Figure 8 shows the proposed encoder, where $|\psi_{j}\rangle$ (j=1,2,…,6) are six input states to be converted into the corresponding logical states $|\psi_{j}\rangle_{L}$. (For simplicity, this figure shows the case of a product state, but this encoder can convert arbitrary input states including entangled states.) This encoder is obtained by adding appropriate CNOT gates enclosed by the dashed rectangle between the Hadamard and CNOT gates in the plus-state encoder in Fig. 2. We determined the added CNOT gates using the following matrix $M_{Z}^{\prime }$ obtained from $M_{Z}$ in Eq. 7 by the Gaussian elimination with $H_{Z}^{\prime }$

$$M_{Z}^{\prime }=\left(\begin{array}{cc} O_{3,12} & M^{\prime }\\ O_{3,15} & M \end{array}\right)$$ (22)

$$M^{\prime }=\left(\begin{array}{cccccccccccccccccc} 1 & 0 & 0 & 1 & 0 & 1 & 1 & 0 & 1 & 0 & 1 & 1 & 0 & 1 & 1 & 0 & 0 & 0\\ 0 & 1 & 0 & 0 & 1 & 0 & 0 & 1 & 1 & 0 & 0 & 1 & 0 & 1 & 1 & 0 & 0 & 0\\ 0 & 0 & 1 & 0 & 0 & 1 & 1 & 0 & 0 & 0 & 0 & 0 & 0 & 1 & 1 & 0 & 0 & 0 \end{array}\right)$$ (23)

where *M* is the matrix in Eq. 8. By its definition, $M_{Z}^{\prime }$ defines logical *Z* operators. Each added CNOT gate corresponds to a pair of 1’s in a row of $M_{Z}^{\prime }$, where one of the pair (the target qubit) corresponds to one of the input qubits (the 13th, 14th, 15th, 28th, 29th, or 30th column) and the other (the control qubit) corresponds to one of the other qubits (one of the 16th to 27th columns). In the following, we explain the validity of this encoder.

**Fig. 8.**
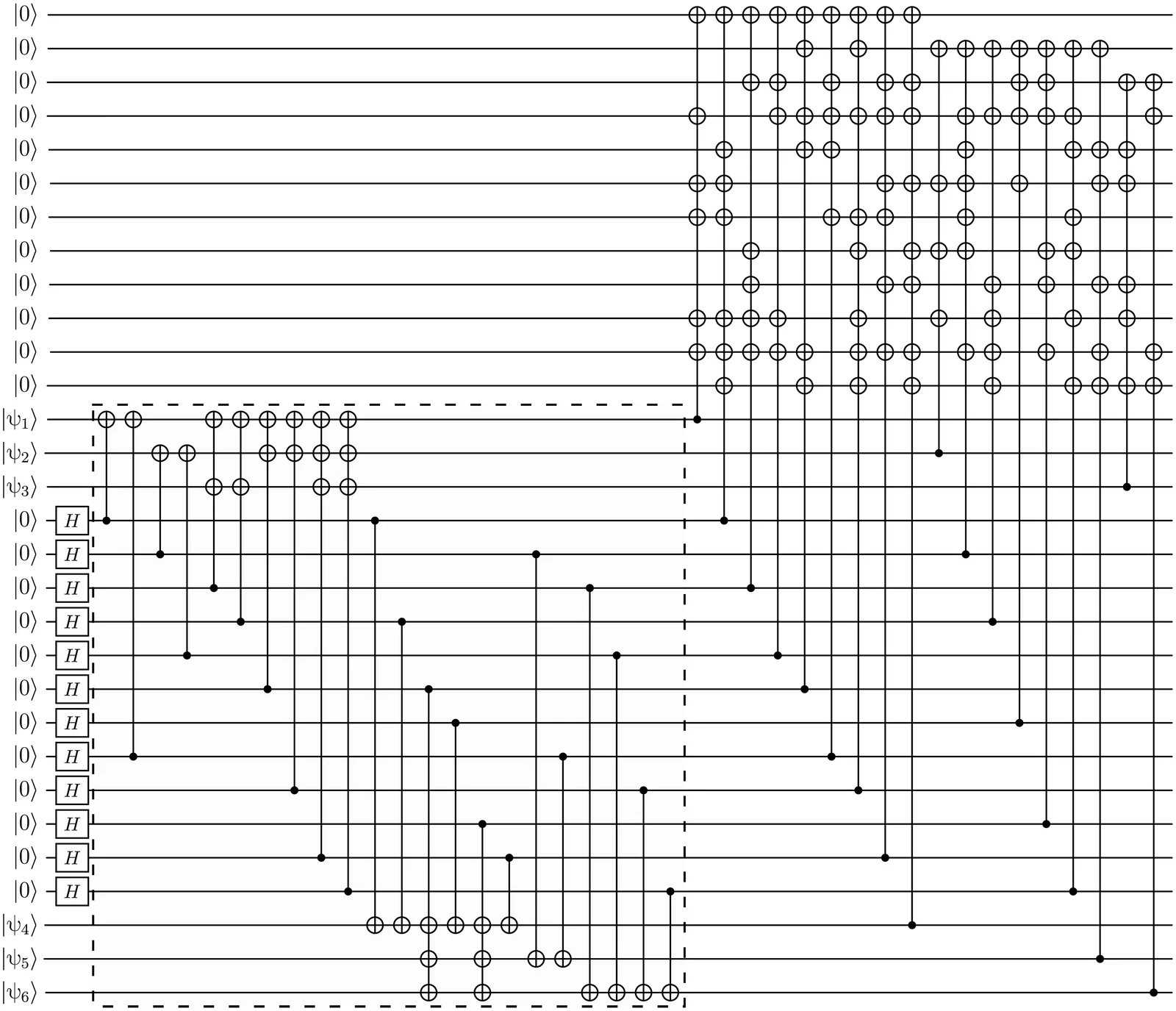
Arbitrary-state encoder for the [[30,6,5]] symplectic double code.

First, when all the input states are ∣+〉, the added CNOT gates does not affect the 30-qubit state, and, consequently, this circuit is equivalent to that in Fig. 2. Thus, we successfully obtain the logical all-plus state $\text{∣+〉}{\;}_{L}^{⊗6}$.

Next, we consider the case where all the input states are ∣−〉 = *Z*∣+〉. Then, we successfully obtain the logical all-minus state $\text{∣−〉}{\;}_{L}^{⊗6}$ as follows

$$\begin{array}{c} U_{\text{CNOT}}U_{\text{CNOT}}^{\prime }\text{∣0〉}{\;}^{⊗12}\text{∣−〉}{\;}^{⊗3}{\text{∣+〉}}^{⊗12}{\text{∣−〉}}^{⊗3}\\ =U_{\text{CNOT}}U_{\text{CNOT}}^{\prime }Z_{13}Z_{14}Z_{15}Z_{28}Z_{29}Z_{30}{\text{∣0〉}}^{⊗12}{\text{∣+〉}}^{⊗18}\\ =U_{\text{CNOT}}Z_{L}^{⊗6}U_{\text{CNOT}}^{\prime }{\text{∣0〉}}^{⊗12}{\text{∣+〉}}^{⊗18}\\ =Z_{L}^{⊗6}U_{\text{CNOT}}{\text{∣0〉}}^{⊗12}{\text{∣+〉}}^{⊗18}\\ =Z_{L}^{⊗6}{\text{∣+〉}}_{L}^{⊗6}\\ ={\text{∣−〉}}_{L}^{⊗6} \end{array}$$ (24)

where $U_{\text{CNOT}}$ and $U_{\text{CNOT}}^{\prime }$ are the unitary operators corresponding to the CNOT gates in Fig. 2 and the added CNOT gates, respectively, and $Z_{L}^{⊗6}$ are the six logical *Z* operators defined by $M_{Z}^{\prime }$. In Eq. 24, we use the following three facts. First, by definition of $M_{Z}^{\prime }$, $U_{\text{CNOT}}^{\prime }Z_{13}Z_{14}Z_{15}Z_{28}Z_{29}Z_{30}=Z_{L}^{⊗6}U_{\text{CNOT}}^{\prime }$. Second, since $U_{\text{CNOT}}^{\prime }$ does not affect its target qubits in ∣+〉, $U_{\text{CNOT}}^{\prime }{\text{∣0〉}}^{⊗12}{\text{∣+〉}}^{⊗18}={\text{∣0〉}}^{⊗12}{\text{∣+〉}}^{⊗18}$. Third, since $U_{\text{CNOT}}$ commutes with $Z_{L}^{⊗6}$, $U_{\text{CNOT}}Z_{L}^{⊗6}=Z_{L}^{⊗6}U_{\text{CNOT}}$. Similarly, any product state of ∣+〉 and ∣−〉 is converted into their corresponding logical states by this encoder.

Finally, from the linearity of unitary operators, this encoder converts any superposition of ∣±〉 product states into its corresponding logical state. Therefore, the quantum circuit in Fig. 8 is the desired arbitrary-state encoder for the [[30,6,5]] symplectic double code.

This arbitrary-state encoder is not fault tolerant. Making it fault-tolerant and evaluating the resulting encoder, e.g., for magic states in a circuit-level noise model, are left for future work.

## DISCUSSION

In this work, we have proposed efficient fault-tolerant encoders for two compact and high-rate symplectic double codes, [[30,6,5]] and [[36,12,5]]. Leveraging these encoders, we have demonstrated low-overhead and low-error-rate logical CNOT gates under a circuit-level noise model. To enable universal quantum computation with the symplectic double codes, we have also developed arbitrary-state encoders for these codes. Further optimizing the encoding circuits for these codes remains an open challenge. The arbitrary-state encoders, together with transversal CNOT and Hadamard gates, enable transversal logical phase gates *S* and non-Clifford gates via state distillation and gate teleportation (*33*, *37*). [More efficient magic state preparation beyond distillation, e.g., using direct measurements of logical operators, which has been realized for the Steane code (*26*), is desired but left as a future challenge]. To achieve addressable logical gates beyond transversal ones, we need fault-tolerant preparation of appropriate logical Clifford states such as partial ∣+〉 states (*33*), which is possible by the arbitrary-state encoders, followed by verification as shown in Fig. 6A. Toward more efficient logical computation, logical gates originating from the *ZX-* duality of the symplectic double code (*25*), such as a fold-transversal CZ gate (*28*) (transversal application of CZ gates between the first and second halves of the code, resulting in a transversal logical CZ gate between the first three and second three logical qubits), will be helpful.

Looking ahead, a critical next step is the experimental implementation of the proposed protocols on physical quantum hardware platforms, such as trapped-ion and neutral-atom systems. The proposed encoders will be helpful for near-term experimental demonstration of low-overhead FTQC with the symplectic double codes. These experiments will also allow us to evaluate the performance of the proposed encoders under realistic noises.

Another interesting direction is scaling up the code size beyond the [[30, 6, 5]] and [[36, 12, 5]] codes, which were chosen because of the suitable sizes for fault tolerance analysis and early experimental realization. Recently, artificial intelligence (AI)–driven discovery of efficient encoding circuits has been reported (*38*). However, the codes have been limited to single–logical qubit ones or high-rate but small-size ones. Toward scaling up, the combination of such an AI-driven approach with the present methods is promising.
