# Supplementary material for: Fault-tolerant quantum computing with high-rate symplectic double codes
Source: Sci Adv. 2026 Nov 13;12(46):eaec4142. doi: 10.1126/sciadv.aec4142 (PMC13763853; doi:10.1126/sciadv.aec4142)
Supplement: Supplementary file 1 [file sciadv.aec4142_sm.pdf]

Science Advances
AAAS

Supplementary Materials for

Fault-tolerant quantum computing with high-rate symplectic double codes

Naoyuki Kanomata and Hayato Goto

Corresponding author: Naoyuki Kanomata, naoyuki.kanomata@riken.jp; Hayato Goto, hayato.goto@riken.jp

Sci. Adv. **12**, eaec4142 (2026)
DOI: 10.1126/sciadv.aec4142

This PDF file includes:

Supplementary Text
Figs. S1 to S5

Supplementary Text

Results for the $[[36, 12, 5]]$ Symplectic Double Code

In the main text, we present the results for the $[[30, 6, 5]]$ symplectic double code. To show that the proposed methods are not limited to this specific code, we provide the corresponding results for the $[[36, 12, 5]]$ symplectic double code in the Supplementary Materials.

The $[[36, 12, 5]]$ symplectic double code is obtained from an $[[18, 6, 5]]$ non-CSS stabilizer code with the following stabilizer matrix (*23*):

$$H = (H_X \mid H_Z) = \left(\begin{array}{c|c}
1\,0\,0\,0\,0\,1\,0\,1\,1\,0\,0\,1\,1\,0\,1\,0\,0\,1 & 0\,0\,0\,0\,0\,0\,0\,0\,1\,0\,0\,0\,0\,1\,0\,1\,0\,0 \\
0\,0\,0\,0\,0\,0\,0\,0\,1\,0\,0\,0\,0\,1\,0\,1\,0\,0 & 1\,0\,0\,0\,0\,1\,0\,1\,0\,0\,0\,1\,1\,1\,1\,1\,0\,1 \\
0\,1\,0\,0\,0\,1\,0\,0\,1\,0\,1\,0\,0\,1\,1\,1\,0\,1 & 0\,0\,0\,0\,0\,0\,0\,0\,1\,1\,0\,0\,1\,0\,0\,0\,0\,1 \\
0\,0\,0\,0\,0\,0\,0\,0\,1\,1\,0\,0\,1\,0\,0\,0\,0\,1 & 0\,1\,0\,0\,0\,1\,0\,0\,0\,1\,1\,0\,1\,1\,1\,1\,0\,0 \\
0\,0\,1\,0\,0\,1\,0\,1\,1\,1\,0\,0\,0\,0\,0\,1\,1\,1 & 0\,0\,0\,0\,0\,0\,0\,0\,1\,1\,1\,0\,1\,0\,0\,1\,0\,0 \\
0\,0\,0\,0\,0\,0\,0\,0\,1\,1\,1\,0\,1\,0\,0\,1\,0\,0 & 0\,0\,1\,0\,0\,1\,0\,1\,0\,0\,1\,0\,1\,0\,0\,0\,1\,1 \\
0\,0\,0\,1\,0\,1\,0\,1\,0\,1\,1\,1\,0\,0\,1\,0\,1\,0 & 0\,0\,0\,0\,0\,0\,0\,0\,1\,1\,1\,1\,0\,0\,0\,1\,1\,0 \\
0\,0\,0\,0\,0\,0\,0\,0\,1\,1\,1\,1\,0\,0\,0\,1\,1\,0 & 0\,0\,0\,1\,0\,1\,0\,1\,1\,0\,0\,0\,0\,0\,1\,1\,0\,0 \\
0\,0\,0\,0\,1\,1\,0\,0\,1\,1\,0\,1\,0\,1\,0\,0\,1\,1 & 0\,0\,0\,0\,0\,0\,0\,1\,0\,0\,0\,0\,0\,1\,0\,1\,1\,1 \\
0\,0\,0\,0\,0\,0\,0\,1\,0\,0\,0\,0\,0\,1\,0\,1\,1\,1 & 0\,0\,0\,0\,1\,1\,0\,1\,1\,1\,0\,1\,0\,0\,0\,1\,0\,0 \\
0\,0\,0\,0\,0\,0\,1\,1\,1\,1\,1\,1\,0\,0\,0\,0\,0\,0 & 0\,0\,0\,0\,0\,0\,0\,0\,0\,0\,0\,0\,1\,1\,1\,1\,1\,1 \\
0\,0\,0\,0\,0\,0\,0\,0\,0\,0\,0\,0\,1\,1\,1\,1\,1\,1 & 0\,0\,0\,0\,0\,0\,1\,1\,1\,1\,1\,1\,1\,1\,1\,1\,1\,1
\end{array}\right).$$

As in the case of the $[[30, 6, 5]]$ code, the corresponding $[[36, 12, 5]]$ symplectic double code is constructed from this matrix according to Eq. (3) in the main text.

By finding six vectors orthogonal to and linearly independent of both H_X and H_Z, we obtain the explicit forms of M_X and M_Z as

$$M_X = M_Z = \begin{pmatrix} M & O_{6,18} \\ O_{6,18} & M \end{pmatrix},$$

where

$$M = \begin{pmatrix}
1\,0\,0\,0\,0\,0\,0\,1\,1\,1\,1\,0\,0\,0\,1\,1\,0\,0 \\
0\,1\,0\,0\,0\,0\,0\,0\,1\,1\,1\,1\,0\,0\,0\,1\,1\,0 \\
0\,0\,1\,0\,0\,0\,1\,0\,0\,1\,1\,1\,0\,0\,0\,0\,1\,1 \\
0\,0\,0\,1\,0\,0\,1\,1\,0\,0\,1\,1\,1\,0\,0\,0\,0\,1 \\
0\,0\,0\,0\,1\,0\,1\,1\,1\,0\,0\,1\,1\,1\,0\,0\,0\,0 \\
0\,0\,0\,0\,0\,1\,1\,1\,1\,1\,0\,0\,0\,1\,1\,0\,0\,0
\end{pmatrix}$$

consists of the above six vectors. The logical Pauli operators are expressed more explicitly as follows:

$$
\begin{aligned}
Z_{L1} &= Z_1Z_8Z_9Z_{10}Z_{11}Z_{15}Z_{16}, & X_{L1} &= X_1X_8X_9X_{10}X_{11}X_{15}X_{16},\\
Z_{L2} &= Z_2Z_9Z_{10}Z_{11}Z_{12}Z_{16}Z_{17}, & X_{L2} &= X_2X_9X_{10}X_{11}X_{12}X_{16}X_{17},\\
Z_{L3} &= Z_3Z_7Z_{10}Z_{11}Z_{12}Z_{17}Z_{18}, & X_{L3} &= X_3X_7X_{10}X_{11}X_{12}X_{17}X_{18},\\
Z_{L4} &= Z_4Z_7Z_8Z_{11}Z_{12}Z_{13}Z_{18}, & X_{L4} &= X_4X_7X_8X_{11}X_{12}X_{13}X_{18},\\
Z_{L5} &= Z_5Z_7Z_8Z_9Z_{12}Z_{13}Z_{14}, & X_{L5} &= X_5X_7X_8X_9X_{12}X_{13}X_{14},\\
Z_{L6} &= Z_6Z_7Z_8Z_9Z_{10}Z_{14}Z_{15}, & X_{L6} &= X_6X_7X_8X_9X_{10}X_{14}X_{15},\\
Z_{L7} &= Z_{19}Z_{26}Z_{27}Z_{28}Z_{29}Z_{33}Z_{34}, & X_{L7} &= X_{19}X_{26}X_{27}X_{28}X_{29}X_{33}X_{34},\\
Z_{L8} &= Z_{20}Z_{27}Z_{28}Z_{29}Z_{30}Z_{34}Z_{35}, & X_{L8} &= X_{20}X_{27}X_{28}X_{29}X_{30}X_{34}X_{35},\\
Z_{L9} &= Z_{21}Z_{25}Z_{28}Z_{29}Z_{30}Z_{35}Z_{36}, & X_{L9} &= X_{21}X_{25}X_{28}X_{29}X_{30}X_{35}X_{36},\\
Z_{L10} &= Z_{22}Z_{25}Z_{26}Z_{29}Z_{30}Z_{31}Z_{36}, & X_{L10} &= X_{22}X_{25}X_{26}X_{29}X_{30}X_{31}X_{36},\\
Z_{L11} &= Z_{23}Z_{25}Z_{26}Z_{27}Z_{30}Z_{31}Z_{32}, & X_{L11} &= X_{23}X_{25}X_{26}X_{27}X_{30}X_{31}X_{32},\\
Z_{L12} &= Z_{24}Z_{25}Z_{26}Z_{27}Z_{28}Z_{32}Z_{33}, & X_{L12} &= X_{24}X_{25}X_{26}X_{27}X_{28}X_{32}X_{33},
\end{aligned}
$$

where X_{Li} and Z_{Li} are the Pauli operators for the i-th logical qubit, and X_j and Z_j are those for the j-th physical qubit.

Supplementary Figures S1–S5 show the results for the $[[36, 12, 5]]$ code, each of which corresponds directly to a figure in the main text. Specifically, Supplementary Fig. S1 corresponds to Fig. 2, Supplementary Fig. S2 to Fig. 3, Supplementary Figs. S3A and S3B to Figs. 5A and 5B, respectively, Supplementary Figs. S4A and S4B to Figs. 7C and 7D, respectively, and Supplementary Fig. S5 to Fig. 8. These results are obtained by applying the proposed methods explained in the main text directly to the $[[36, 12, 5]]$ code. As shown in Fig. 4, the logical-CNOT error probabilities for the $[[36, 12, 5]]$ code are comparable to those for the $[[30, 6, 5]]$ code with a reasonable increase in qubit resources. This supports the generality of the proposed methods.

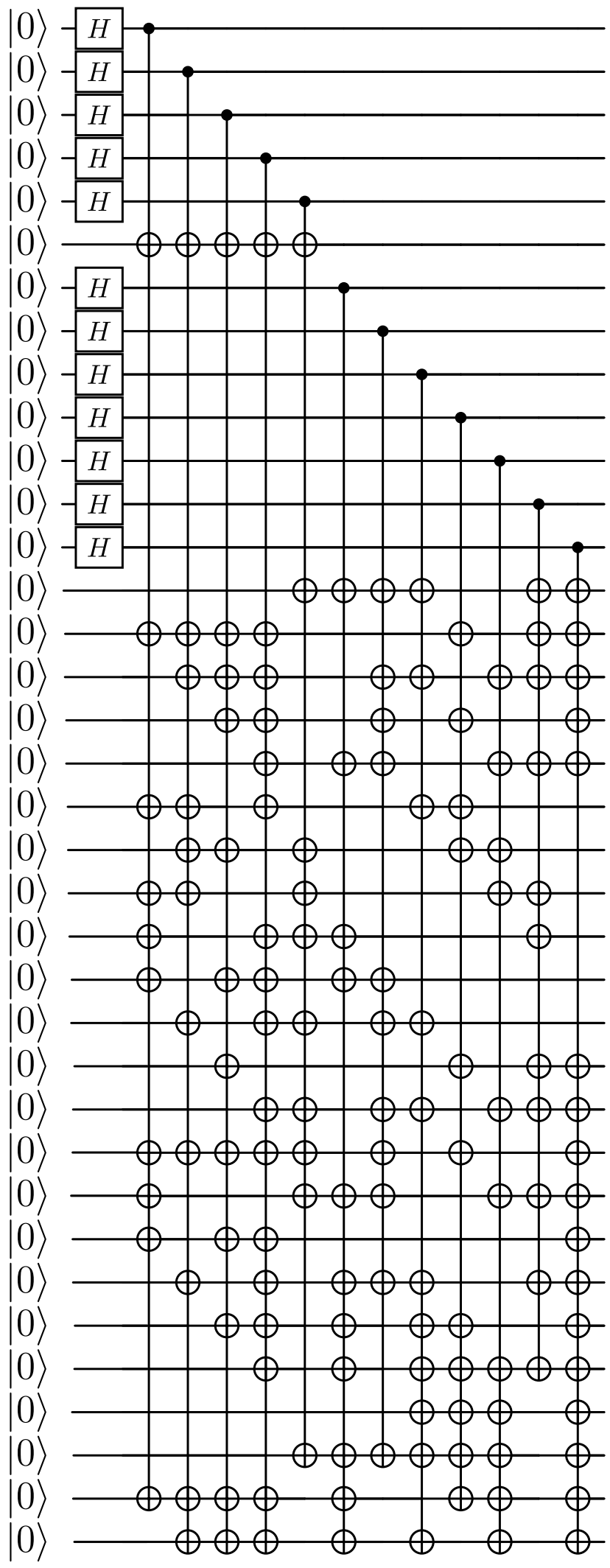

Fig. S1. **Zero-state encoder for the $[[36, 12, 5]]$ symplectic double code with 143 CNOT gates.**

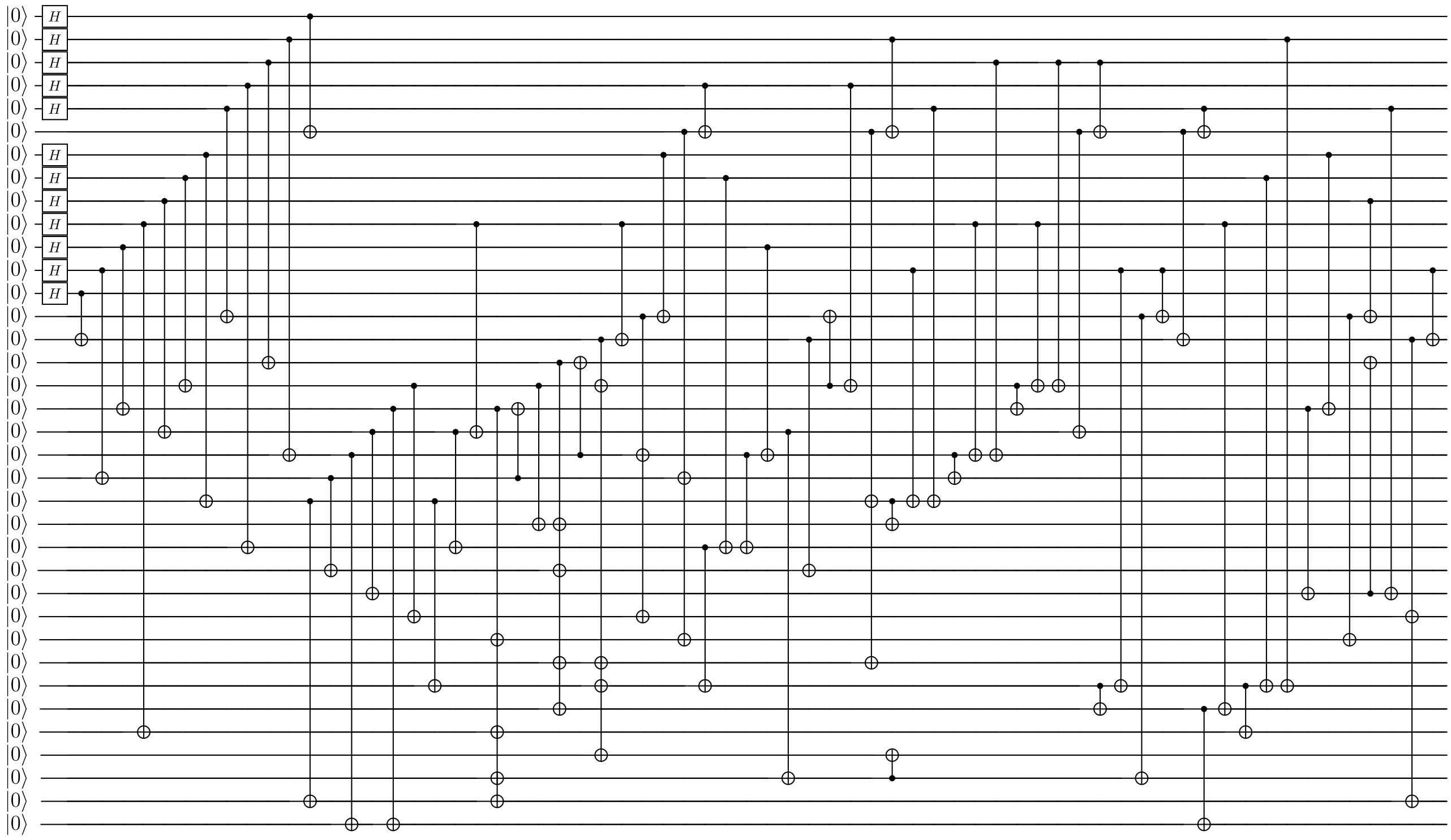

Fig. S2. **Zero-state encoder for the $[[36, 12, 5]]$ symplectic double code with 86 CNOT gates.**

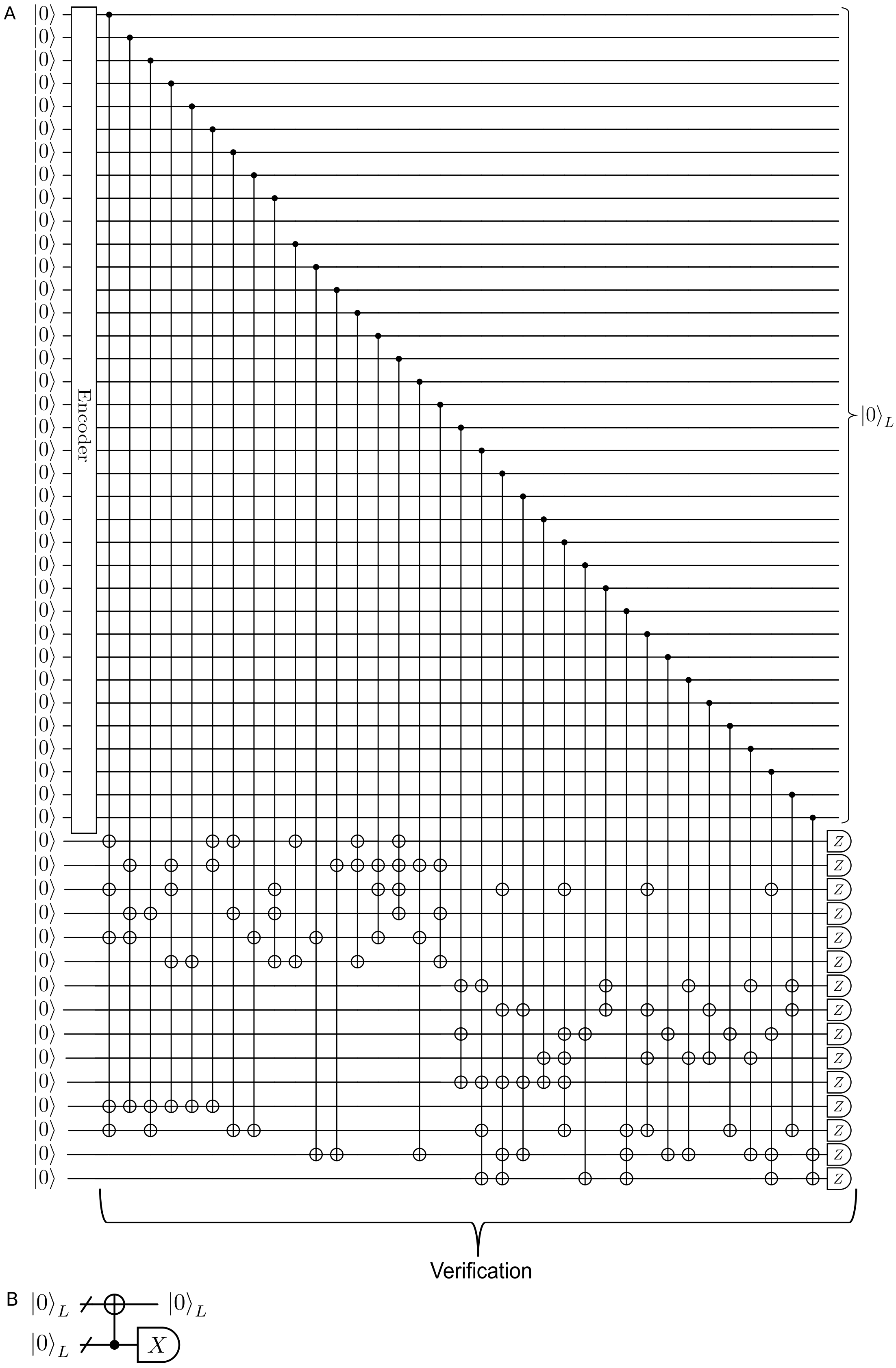

Fig. S3. **Fault-tolerant zero-state encoder for the** $[[36, 12, 5]]$ **symplectic double code.** (A) Zero-state encoder utilizing Goto's verification method (*26*) to eliminate harmful X errors. The "Encoder" box is the quantum circuit shown in Fig. S2. (B) Verification procedure to eliminate harmful Z errors. The two input states are prepared by the encoder in (A).

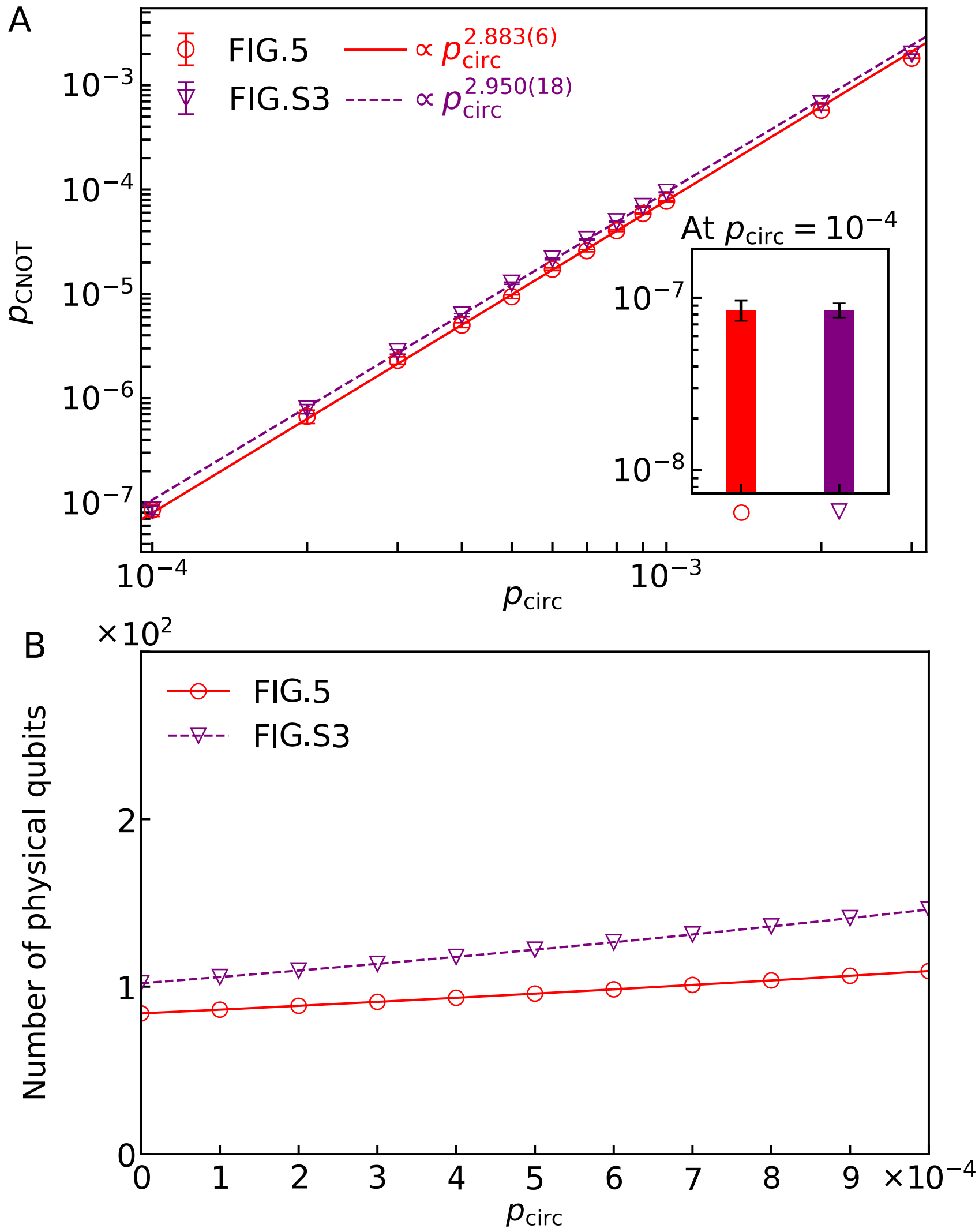

Fig. S4. **Evaluation of the encoders and logical CNOT gates for the [[36, 12, 5]] symplectic double code.** (A) Numerical results of the logical-CNOT error probability p_{CNOT} as a function of the error rate, p_{circ}, of the circuit-level noise model. The circles and triangles show the results for the [[30, 6, 5]] and [[36, 12, 5]] symplectic double codes, respectively, where the proposed encoders in Figs. 5 and S3 are used, respectively. The scaling exponent for the [[36, 12, 5]] code was obtained by using the data over the range of p_{circ} from 10^{-4} to 10^{-3}. The results for the [[30, 6, 5]] code are the same as those in Fig. 7C in the main text. (B) Average total number of physical qubits required for preparing a logical all-plus/all-zero state passing the verification stage with no detected errors.

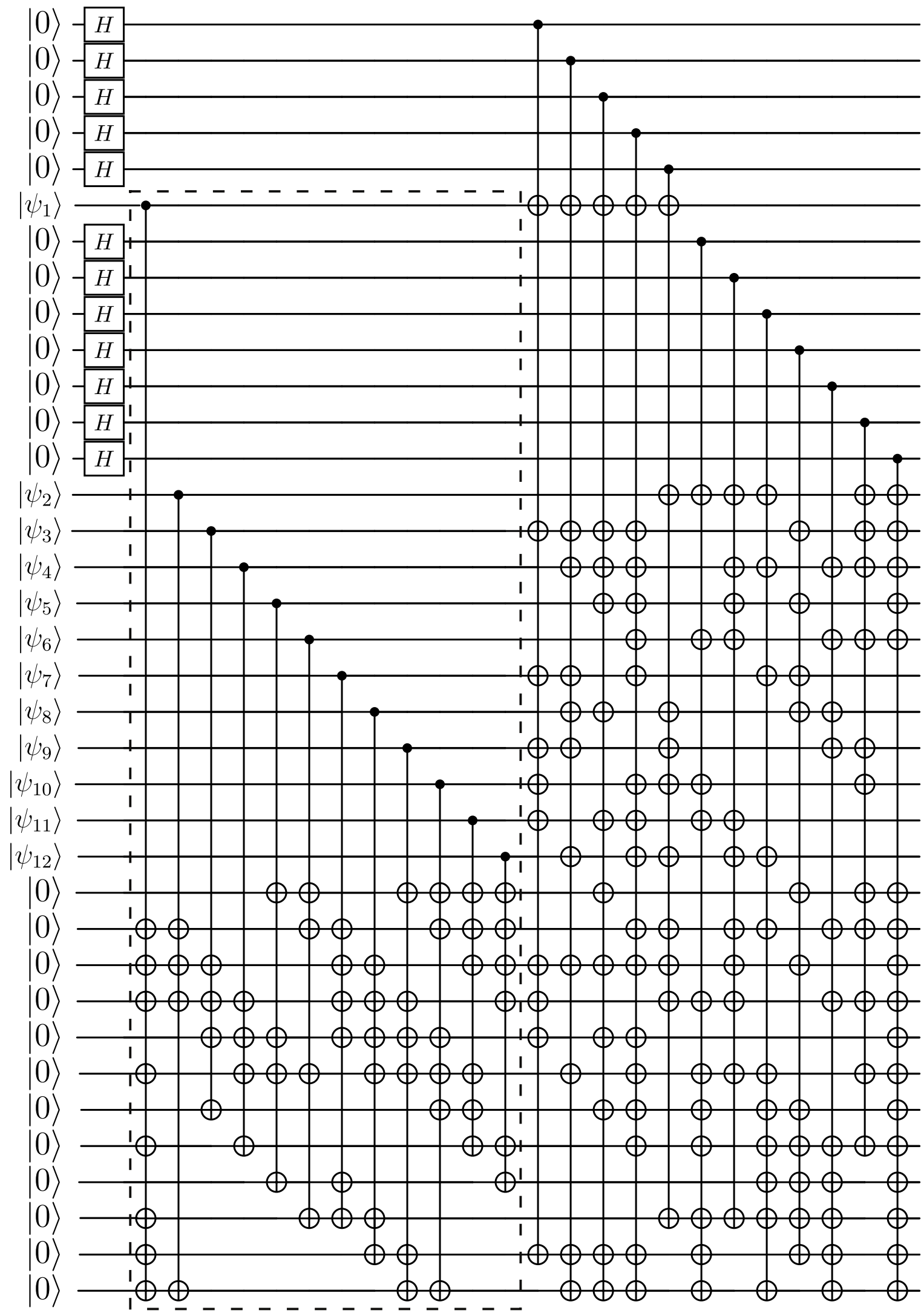

Fig. S5. **Arbitrary-state encoder for the** $[[36, 12, 5]]$ **symplectic double code.**
